# Rabies in Captive Deer, Pennsylvania, USA, 2007–2010

**DOI:** 10.3201/eid1801.111189

**Published:** 2012-01

**Authors:** Brett W. Petersen, Danielle M. Tack, Allison Longenberger, Aliza Simeone, Mària E. Moll, Marshall P. Deasy, Jesse D. Blanton, Charles E. Rupprecht

**Affiliations:** Centers for Disease Control and Prevention, Atlanta, Georgia, USA (B.W. Petersen, D.M. Tack, A. Longenberger, J.D. Blanton, C.E. Rupprecht);; Pennsylvania Department of Agriculture, Harrisburg, Pennsylvania, USA (A. Simeone);; Pennsylvania Department of Health, Harrisburg (M.E. Moll, M.P. Deasy)

**Keywords:** rabies, epidemiology, lyssavirus, deer, Pennsylvania, viruses, zoonoses

## Abstract

Since January 2007, a total of 11 rabid deer from 4 deer farms have been identified in 2 neighboring Pennsylvania counties. Vaccination of deer against rabies, decreasing wildlife animal contact with deer, and education of deer farmers may prevent further cases of rabies in captive deer and exposures to humans.

Rabies is an acute progressive encephalitis caused by highly neurotropic zoonotic lyssaviruses. The disease is nearly always fatal in humans if postexposure prophylaxis (PEP) is not administered promptly after exposure (*1**,**2*). Although human cases of rabies are rare in the United States, each year nearly 7,000 rabid animals are reported and ≈35,000 humans receive PEP (*3**,**4*).

Since January 2007, a total of 11 rabid deer from 4 deer farms were identified in the neighboring counties of Lancaster and Chester, Pennsylvania, USA. These farms were located with a 45-mile radius in an area of high prevalence for rabies among all animals (excluding bats) (Figure 1). We investigated this cluster to identify factors that might have contributed to disease transmission, assess the risk to humans, and provide recommendations for prevention.

**Figure 1:**
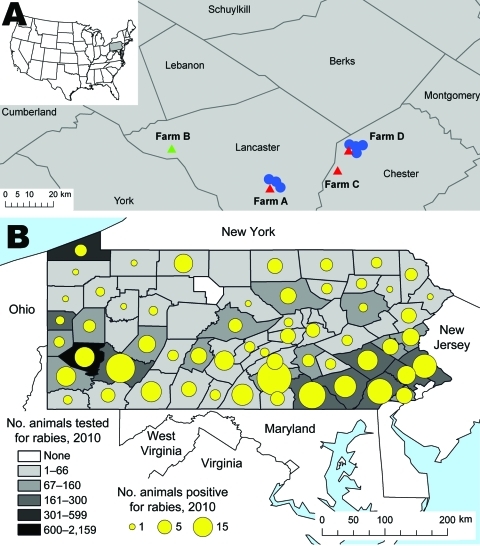
Reports of suspected and confirmed rabies among all animals (excluding bats) in Pennsylvania, 2010.

## The Study

**Figure 2:**
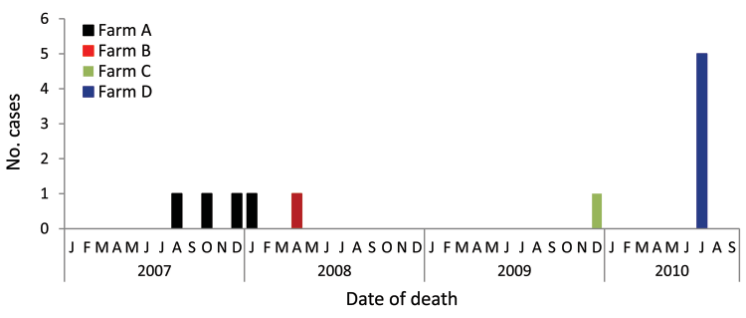
Rabies deaths among captive deer at 4 farms in Lancaster and Chester counties, Pennsylvania, USA, 2007–2010.

The death of a rabid captive deer, an adult doe, was reported initially in August 2007 at farm A; during October 2007–January 2008, three buck fawns from this farm also died of rabies (Figure 2). In April 2008 and December 2009, two adult does died of rabies at farm B and farm C, respectively (Figure 2). Lastly, during July 2010, one adult doe, followed by 4 buck fawns, died at farm D (Figure 2). All reported cases were laboratory confirmed, and diagnostic testing detected a rabies virus variant associated with raccoons.

We conducted a case–control study among deer farms in Pennsylvania. A case farm was defined as a registered deer farm with >1 laboratory-confirmed case of rabies in a deer. A control farm was defined as a registered deer farm that did not report any laboratory-confirmed rabies in deer. Ten control farms were chosen by referral from farmers and from the Pennsylvania Deer Farmers Association website. Control farms were limited to the affected counties and 1 noncontiguous county to account for local ecologic diversity. Four farms (farms A–D) met the case definition. Staff from the Centers for Disease Control and Prevention, state and local health departments, and the Pennsylvania Department of Agriculture conducted site visits to each affected farm. A standardized questionnaire was administered in person or by telephone to all study farms during August 2–5, 2010. Proportions were compared with Fisher exact test by using SAS version 9.2 (SAS Institute Inc., Cary, NC, USA).

Farmers’ ages, levels of education, and number of years farming did not differ between case and control farms (Table 1). Control farms tended to be larger than case farms (27.4 vs. 15.3 acres; odds ratio [OR] 0.99, 95% CI 0.95–1.03) with more deer per acre (36.8 vs. 34.7; OR 0.99, 95% CI 0.96–1.04), although neither difference was significant (Table 1). Farming practices did not differ between case and control farms (Table 1). Trough and bottle feeding of deer were common practices, and most (71%) farms reported using sweet deer feed containing molasses, which might attract rabies reservoir species, such as raccoons. Deer were moved infrequently between pens, and few outside deer were brought onto the farms (mean 2.5 deer/year); however, interstate travel of deer was reported. Most (71%) farms reported vaccinating deer against at least 1 disease, but deer were vaccinated on case farms only in response to previously rabid deer (Table 1). A low perceived risk for rabies was cited as the primary barrier to rabies vaccination among control farms. In contrast, witnessed contact between deer and rabies reservoir species was relatively common (43% of farms reported contact with skunks, and 36% reported contact with raccoons) (Table 2).

**Table 1:** Characteristics and practices of farms with rabid deer, Pennsylvania, USA, 2007–2010

Characteristic/practice	Case farms, n = 4	Control farms, n = 10	Total, n = 14	Odds ratio (95% CI)
Mean age of farmer, y	41.3	46.6	45.1	0.96 (0.88–1.10)
Farmer education, some high school or above, no. (%); reference = some high school or below	1 (25)	5 (50)	6 (43)	0.33 (0.03–4.40)
Mean time farming, y (range)	10.8 (5–17)	9.3 (2–17)	9.7 (2–17)	1.1 (0.83–1.37)
Mean size of farm, acres (range)	15.3 (2–45)	27.4 (3–170)	23.9 (2–170)	0.99 (0.95–1.03)
No. deer on farm, mean (range)	69.8 (13–159)	75.8 (16–230)	74.1 (13–230)	0.99 (0.98–1.02)
No. deer/acre in smallest pen, mean (range)	34.7 (10.7–90.9)	36.8 (8–89.3)	36.2 (8–90.0)	0.99 (0.96–1.04)
No. deer brought onto farm annually, mean	1.25	3.0	2.5	0.67 (0.23–1.92)
Farms reporting deer vaccination, no. (%)	4 (100)	7 (70)	11 (79)	0.78 (0.06–10.86)
Farms reporting deer vaccinated against rabies, no. (%)	4 (100)	0	4 (28)	Not calculated
Farms reporting feeding method, no (%)				
Trough	4 (100)	9 (90)	13 (93)	Not calculated
Bottle	2 (50)	9 (90)	11 (79)	0.11 (0.01–1.91)
Tube feeder	1 (25)	3 (30)	4 (29)	0.78 (0.06–10.86)
Farms reporting use of sweet feed, no. (%)	3 (75)	7 (70)	10 (71)	1.29 (0.09–17.95)
Farms reporting deer contact with rabies reservoir species, no. (%)				
Skunks	2 (50)	4 (40)	6 (43)	1.5 (0.15–15.46)
Raccoons	2 (50)	3 (30)	5 (36)	2.3 (0.22–25.25)
Foxes	0	1 (10)	1 (7)	Not calculated

**Table 2:** Rabies knowledge, exposures, and health-seeking behavior among deer farmers, Pennsylvania, USA, 2007–2010

Variable	No. (%) respondents			Odds ratio (95% CI)
	Case farm, n = 4	Control farm, n = 10	Total, n = 14	
Self-reported knowledge of rabies, basic; reference = advanced	1 (25)	9 (90)	10 (71)	0.04 (0.002–0.79)
Farms reporting human rabies vaccination	4 (100)	0	4 (29)	Not calculated
Farms reporting an animal bite on farm	0	4 (40)	4 (29)	Not calculated
Farms reporting an animal scratch on farm	2 (50)	5 (50)	7 (50)	1.0 (0.1–10.17)
Farms reporting bare skin contact with animal saliva	4 (100)	10 (100)	14 (100)	Not calculated
Farms reporting bare skin contact with animal tissue	2 (50)	2 (20)	4 (29)	4.0 (0.33–48.66)
Advocated action if exposed to rabies virus				
Seek medical care	3 (75)	7 (70)	10 (71)	1.29 (0.09–17.95)
Get rabies vaccination	2 (50)	4 (40)	6 (43)	1.50 (0.15–15.46)
Call county health department	1 (25)	0	1 (7)	Not calculated
Have animal tested for rabies	0	1 (10)	1 (7)	Not calculated
Wash with soap and water	0	0	0	Not calculated

Each of the 4 deer farmers from case farms received rabies PEP because of exposures to the rabid deer. Potential sources of exposure were common. All deer farmers reported bare skin contact with animal saliva, 50% reported being scratched by an animal, 29% reported being bitten by an animal, and 29% reported bare skin contact with animal tissue (Table 2). Case farms had significantly higher self-reported knowledge about rabies, probably because of their direct experience with the disease (Table 2). However, knowledge of rabies among control farms was low (90% of farmers reported knowledge as basic), and none of the farmers indicated that they should wash with soap and water if potentially exposed to the rabies virus (Table 2).

## Conclusions

Since 1990, a total of 104 rabid deer have been reported in the United States. However, to the best of our knowledge, the cases in this report are the only reported cases in captive deer. The number of captive deer in the United States is increasing because of the growth of the deer farming industry. In Pennsylvania alone, ≈1,200 deer farms currently operate in 63 of the 67 counties (*5*). Without appropriate prevention and control efforts integrating the concepts of One Health (*6*), rabies in captive deer has the potential to threaten human and animal health.

The primary method of preventing rabies in animals is vaccination, which in turn reduces the risk for transmission to humans. Although not licensed for use in deer, rabies vaccine is expected to have a similar safety and efficacy profile in deer as it does in other ruminants. The Compendium of Animal Rabies Prevention and Control recommends that rabies vaccination be considered in livestock that have frequent contact with humans or in livestock that are particularly valuable (*6*). Captive deer would meet both criteria on the basis of the human exposures reported in the survey and their economic value (individual deer can cost hundreds to hundreds of thousands of dollars). Interstate transportation of deer is state regulated and generally prohibits importation of deer from states with endemic chronic wasting disease. However, the survey did document interstate travel of captive deer, which raises concern for the possible translocation of a raccoon rabies virus variant across oral rabies vaccination boundaries. To decrease this risk, rabies vaccination should be strongly considered in any deer transported between farms or across state borders.

Although no specific management practices were identified as major risk factors among affected deer farms, the rabies virus most likely was transmitted to the deer through contact with wildlife, as reported in the survey. However, deer-to-deer transmission could not be excluded at farms where multiple deer were affected (i.e., farms A and D). At both locations, adult does died, followed by buck fawns, suggesting that infection could have occurred during close maternal activities involving saliva transmission. However, not all of the rabid fawns belonged to the rabid does, and some of the rabid fawn’s mothers remained healthy, even though doe can display maternal behavior toward multiple fawns. Regardless, measures to decrease contact between captive deer and rabies reservoir species should be implemented. Such measures might include trapping and removing such species, eliminating brush, groundhog burrows, or other potential sources of dens or cover for terrestrial carnivores; avoiding planting crops or storing food near deer pens; and using elevated, closed feeders (such as tube feeders) placed away from pen fences.

The survey also demonstrated that animal exposures, such as contact of bare skin with animal saliva, commonly occur on deer farms, and provide a potential route of transmission in the presence of any open cuts or wounds. This point is particularly important given deer farmer knowledge about rabies appears limited, especially with regard to exposures. Although 71% of respondents indicated knowing they should seek medical care if exposed to the rabies virus, none indicated they should wash with soap and water. These findings provide evidence of the danger of rabies virus transmission to humans from captive deer and the need to educate deer farmers.
